# Characterization of Aroma Active Compounds in Fruit Juice and Peel Oil of Jinchen Sweet Orange Fruit (*Citrus sinensis* (L.) Osbeck) by GC-MS and GC-O

**DOI:** 10.3390/molecules13061333

**Published:** 2008-06-12

**Authors:** Yu Qiao, Bi Jun Xie, Yan Zhang, Yun Zhang, Gang Fan, Xiao Lin Yao, Si Yi Pan

**Affiliations:** College of Food Science and Technologhy, Huazhong Agricultural University, Shizhishan Street No. 1, Wuhan, Hubei, 430070, P. R. China

**Keywords:** Aroma, GC-MS, GC-O, Jinchen, fruit juice, peel oil

## Abstract

Gas chromatography-mass spectrometry (GC-MS) and gas chromatography-olfactometry (GC-O) were used to determine the aromatic composition and aroma active compounds of fruit juice and peel oil of Jinchen sweet orange fruit. Totals of 49 and 32 compounds were identified in fruit juice and peel oil, respectively. GC-O was performed to study the aromatic profile of Jinchen fruit juice and peel oil. A total of 41 components appeared to contribute to the aroma of fruit juice and peel oil. Twelve components were the odorants perceived in both samples. The aromatic compositions of fruit juice were more complex than that of peel oil. Ethyl butanoate, β-myrcene, octanal, linalool, α-pinene, and decanal were found to be responsible for the aromatic notes in fruit juice and peel oil. Nineteen components have been perceived only in the juice and ten compounds were described as aromatic components of only the peel oil by the panelists. These differences lead to the different overall aroma between fruit juice and peel oil.

## Introduction

Orange fruits are the most popular ones for consumers throughout the world due to their pleasant flavors and nutritional value [1]. The fruits are both consumed fresh and industrially processed. The pulps, which are rich in soluble sugars, significant amounts of vitamin C, pectin, fiber and different organic acids, are mainly used to process into juice. Orange peels containing abundant fragrant substances are extensively applied for processing into essential oils which are used commercially for flavoring foods, beverages, perfumes, cosmetics, etc.

Analytical research on the aroma compounds in orange fruit has been carried out for many years. Many researchers have studied the volatile compounds of this fruit using different analytical methods [2,3,4,5,6,7]. As a result of these studies, more than 200 components have been described as components of the flavor of sweet orange, and the major aroma compounds found in orange juice are hydrocarbons, alcohols, aldehydes, esters, and ketones. The presences of terpenes with traces of oxygenated components are the aromatic character in the orange essential oil and peel oil [8,9,10,11,12,13].

Only a small part of all volatiles present in food has a primary impact on aroma [14]. The aromatic quality of the fruit is primarily dependent on the aroma active compounds. Gas chromatography-olfactometry (GC-O) analysis is a valuable method for the detection of aroma active compounds from a food matrix [15,16,17]. The three main GC-O methods are the dilution analysis, detection frequency and perceived intensity method [18,19], and these techniques have been employed by various researchers to determine the aroma impacting compounds in orange juice [20,21,22,23]. Solid phase microextraction (SPME) is a rapid, solvent-free, low-cost, simple extracting method [24,25], and usually combined with GC-O analysis. The amounts of flavor compounds absorbed on the SPME coating were greatly influenced by the diffusion of flavor components through orange juice into the headspace and the headspace gas reach to the SPME coating. Therefore, the high correlations between the concentrations and GC peak area were not obtained for all the volatile compounds [26]. It is also difficult to perform the dilution analysis such as AEDA using SPME extracts. Kim *et al*. varied the GC injector split ratio to achieve dilution stepwise of the flavor compounds in HS-SPME–GC-O using an aqueous model system and yuzu tea. The research only demonstrated that serial dilution of the four flavor compounds in yuzu can be achieved successful through the increase of GC split ratio, other aroma compounds was not be mentioned [27]. Some researchers conducted the intensity method or frequency method combined with SPME to analysis orange juice flavor. Bazeome *et al*. stated that ethyl butanoate, mycrene, *trans*-2-nonenal, decanal, octanal, terpinen-4-ol, *cis*-hex-3-enal and three unkown compounds were aroma active compounds in unpasteurized juice and heated juice, using SPME extraction and intensity method [20]. Arena *et al*. used frequency detection method and SPME to find twenty-two odor active compounds in hand-squeezed juices of Moro, Tarocco, Washington navel and Valencia late oranges [23].

GC–O procedure has also been conducted to characterize the aroma active components in some citrus essential oils. Lin and Rouseff detected 38 aroma-active compounds in commercial cold-pressed grapefruit oil using a time–intensity GC-O procedure and concluded that the greatest aroma intensity compounds were1, 8-cineole, octanal, dodecanal, *trans*-4,5-epoxy-(*E*)-2-decenal, β-sinensal and nootkatone [28]. Högnadóttir and Rouseff found 55 aroma active compounds of the detected 95 volatile compounds in orange essence oil. The flavor compounds in orange peel oil extracted by SPME have never been reported [11].

Jinchen is a native sweet crop of *Citrus Sinensis*. It originated from the Sichuan province and has been widely planted in the middle and upper regions of the Yangtze River. No research on Jinchen aroma has ever been reported, though Jinchen is of economic importance to the citrus industry in China. The aim of the study was to determine the aroma active compounds in Jinchen orange fruit juice and peel oil by GC-MS and GC-O, as well as to provide useful information about the most important potent odorants in this fruit.

## Results and Discussion

### Volatile components in fresh Jinchen fruit juice and peel oil

In the analysis of volatile components present in Jinchen fruit juice, a total of 49 compounds were identified including 20 hydrocarbons, 10 aldehydes, nine esters, nine alcohols, and one ketone (Table 1). Limonene, followed by linalool, terpinen-4-ol, β-myrcene, α-terpineol, octanal, and γ-terpinene were the components found in greatest proportions.

**Table 1:** Volatile components identified in fruit juice and peel oil of Jinchen fruit by GC-MS.

RI (HP-5)	RIL	Compound name	Peak area (%) ± SD		Identification
			Fruit juice	Peel oil	
600	613 [29]	Ethyl acetate	0.02±0.00	n.d.	MS, RT
710	712 29	Ethyl propanoate	0.01±0.01	n.d.	MS, RI
725	724 29	Methyl butanoate	trace	n.d.	MS, RI
794	806 29	Hexanal	0.07±0.06	n.d.	MS, RT
796	800 11	Ethyl butanoate	0.24±0.03	n.d.	MS, RT
847	848 11	*trans*-2-Hexenal	0.05±0.00	n.d.	MS, RT
900	910 29	Heptanal	trace	n.d.	MS, RI
924	923 30	α-Thujene	0.03±0.02	0.04±0.00	MS, RI
930	934 11	α-Pinene	0.61±0.10	0.76±0.02	MS, RT
958	968 29	Benzaldehyde	0.04±0.01	n.d.	MS, RT
971	974 11	Sabinene	0.02±0.01	0.49±0.01	MS, RI
973	990 11	β-Pinene	0.07±0.01	n.d.	MS, RI
991	994 11	β-Myrcene	2.38±0.33	1.88±0.04	MS, RT
1004	1005 15	Octanal	1.36±0.13	0.34±0.09	MS, RT
1010	1012 11	δ-3-Carene	n.d.	0.14±0.07	MS, RI
1007	1039 15	Limonene	77.8±4.86	90.85±0.11	MS, RT
1053	1036 10	*cis*-β-Ocimene	0.28±0.02	0.26±0.03	MS, RI
1063	1074 11	γ-Terpinene	0.92±0.23	1.21±0.03	MS, RI
1074	1074 11	1-Octanol	0.04±0.02	n.d.	MS, RT
1089	1096 11	Terpinolene	0.18±0.02	0.08±0.00	MS, RI
1104	1107 11	Linalool	3.9±0.82	0.92±0.02	MS, RT
1106	1109 11	Nonanal	n.d.	0.08±0.01	MS, RT
1133	1133^29^	Ethyl 3-hydroxy-hexanoate	0.08±0.01	n.d.	MS, RI
1135		(±)-4-Acetyl-1-methyl-cyclohexene	0.03±0.01	n.d.	MS
1139	1147 11	*trans*-Limonene oxide	n.d.	0.01±0.00	MS, RI
1149	1159 11	β-Terpineol	0.15±0.11	n.d.	MS, RI
1159	1159 11	Citronellal	n.d.	0.06±0.00	MS, RI
1185	1179 15	Terpinen-4-ol	3.06±0.80	n.d.	MS, RI
1199	1207 11	α-Terpineol	1.61±0.45	0.07±0.01	MS, RT
1208	1208 15	Decanal	0.51±0.05	0.21±0.01	MS, RT
1223	1229 11	*trans*-Carveol	0.1±0.03	n.d.	MS, RI
1232	1237 31	Nerol	0.19±0.16	n.d.	MS, RI
1244	1248 29	Neral	0.02±0.00	0.06±0.02	MS, RT
1247	1256 11	Carvone	0.14±0.04	n.d.	MS, RI
1258	1252 11	Geraniol	0.04±0.01	n.d.	MS, RI
1273	1280 29	Geranial	0.03±0.01	0.11±0.03	MS, RT
1277	1290 11	Perillaldehyde	0.33±0.11	0.03±0.00	MS, RI
1295	1303 29	Thymol	0.03±0.01	trace	MS, RI
1307	1314 29	Undecanal	0.02±0.00	n.d.	MS, RI
1338		*cis*-Carveyl acetate	trace	n.d.	MS
1350	1347 11	α-Cubebene	0.02±0.00	n.d.	MS, RI
1354	1348 11	Citronellyl acetate	0.02±0.00	0.01±0.00	MS, RT
1365	1376 11	Neryl acetate	0.04±0.01	0.02±0.00	MS, RT
1377	1380 29	α-Copaene	0.05±0.01	0.04±0.00	MS, RI
1384	1385 29	Geranyl acetate	0.03±0.01	trace	MS, RT
1391	1390 30	β-Cubebene	0.01±0.00	0.03±0.01	MS, RI
1393	1393 30	β-Elemene	0.02±0.00	n.d.	MS, RI
1417	1411 11	Dodecanal	n.d.	0.03±0.01	MS, RI
1412	1427 30	β-Gurjurene	n.d.	0.01±0.00	MS, RI
1450	1456 30	Alloaromadendrene	trace	n.d.	MS, RI
1455	1454 30	α-Caryophyllene	0.01±0.00	n.d.	MS, RI
1482	1490 29	Germacrene D	0.02±0.01	0.08±0.01	MS, RI
1485		2-Isopropenyl-4a,8-dimethyl-1,2,3,4,4a, 5,6,7-octahydronaphthalene	0.02±0.00	n.d.	MS
1495	1498 29	Valencene	0.38±0.03	0.05±0.01	MS,RT
1499	1491 31	Bicyclogermacrene	n.d.	trace	MS,RI
1525		δ-Amorphene	0.09±0.01	0.05±0.01	MS
1812	1833 11	Nootkatone	0.02±0.01	0.01±0.00	MS,RI
		Total	95.05±8.43	97.94±0.55	

RT: identified by retention time of standard compounds RI: identified by retention index and compared with those reported in the literature MS: confirmed by mass spectrum n.d.: not detected

In peel oil extracts, a total of 32 compounds were identified in the volatile fraction, including 16 hydrocarbons and eight aldehydes, three esters, three alcohols, one ketone and one terpenic oxide(Table 1). Among these components, limonene was the one found in greatest concentrations, representing 90.85% of all volatiles. Other predominant components were β-myrcene, γ-terpinene, linalool and α-pinene.

In both samples the major compounds were monoterpenes and sesquiterpenes. The peel oil contained terpenes at higher levels than those of the fruit juice, except for *cis*-β-ocimene, β-myrcene, terpinolene, δ-amorphene and valencene. Three sesquiterpenes which could not be found in peel oil were detected in small quantity in fruit juice: α-cubebene, β-elemene, and α-caryophyllene. Bicyclogermacrene, which had been reported in lime oils by Tu *et al*. [9] and Ferhat *et al*. [31] was found in Jinchen peel oil in minor amounts (0.01%), but was not found in Jinchen fruit juice.

It is interesting to note the large difference between the numbers of alcohols and esters found in the fruit juice and peel oil. Six alcohols only found in the fruit juice were 1-octanol, β-terpineol, terpinen-4-ol, nerol, *trans*-carveol, and geraniol. These have been mentioned as important compounds associated to the pulp in the juice [32]. Another possible reason for their presence is that the alcohols are reaction products generated during the processing or storage of the orange juice. Terpinen-4-ol is the degradation product of D-limonene and linalool during the heat treatment of juice and its concentration significantly increased during the storage of the heat-treated juices [33].

Esters have been described to be most important to orange flavor. Ethyl butanoate followed by ethyl acetate, ethyl propanoate, and methyl butanoate, only present in fruit juice, were not detected in peel oil. These lower boiling points and high volatility esters could have higher degree of affinity in the pulp. Aldehyde content, especially of octanal and decanal, is generally considered one of the standards for characterization for orange peel oil, [2]. These compounds were the most predominant among the aldehyde compounds in both samples, occurring in higher proportions in fruit juice than in peel oil. In addition, other aliphatic aldehydes play an important role in orange flavor [11]. Nonanal, and dodecanal were only present in peel oil, whereas hexanal, *trans*-2-hexenal, heptanal, undecanal were only detected in fruit juice. A terpenic aldehyde only present in the peel oil was citronellal, constituting 0.06% of all volatile compounds, which has been reported in many citrus oils [9,11,13,28]. The aldehydes are most likely enzymatic degradation products of unsaturated fatty acids such as oleic acid, linoleic acid and linolenic acid [28].

With respect to the rest of the identified components, nootkatone was found in both fruit juice and peel oil. It is a typical component of grapefruits, and its content in the fruits increases with maturation and storage [34]. One terpenic oxide compound (*trans*-limonene oxide) was identified only in the peel oil of Jinchen fruit. It is possible that the limonene oxidation during the process and storage may be responsible for the formation of this compound in peel oil.

### Gas chromatography-/olfactometry (GC-/O) analysis

It was cosidered very necessary to determine the components responsible for the quality of the aroma of Jinchen fruit, therefore, GC-O was performed for further studying the aromatic profile of Jinchen fruit juice and peel oil.

The odor descriptors for all volatile components perceived by panelists in fruit juice and peel oil extracts of Jinchen fruit are shown in Table 2. As a result of the different odor thresholds of each component, not all of the compounds quantified contribute to the aroma in the juice and peel oil. However, olfactometric analysis utilizes the highly selective detector (human nose) that can detect potent low-level aroma-active compounds which could not be quantified by GC-MS (*trans*-2-hexen-1-ol, ethyl 3-hydroxyhexanoate, and six unknown compounds).

**Table 2:** Aroma active compounds of fruit juice and peel oil detected by GC-O.

Compound name	RI(HP-5)	Odor	Intensity±SD	
			Fruit juice	Peel oil
Hexanal	794	Green	2.3 ± 0.9	n.d.
Ethyl butanoate	795	Fruity, Apple	3.8 ± 0.5	n.d.
*trans*-2-Hexenal	849	Green	1.8 ± 0.4	n.d.
*trans*-2-Hexen-1-ol	869	Green	2.6 ± 0.5	n.d.
α-Pinene	930	Piney	3.3 ± 0.9	3.6 ± 0.5
Camphene	947	Camphor	n.d.	1.0 ± 0
β-pinene	982	Piney	3.5 ± 0.6	n.d.
NI	984	Grassy	3.3 ± 0.5	n.d.
β-Myrcene	992	Lemon, Grapefruit, Musty, Spicy	3.6 ± 0.8	3.3 ± 0.5
Octanal	1011	Fruity, Citrusy	3.8 ± 0.4	2.9 ± 0.9*
Limonene	1040	Lemon, Minty, Orange	2.7 ± 0.8	2.5 ± 0.8
γ-Terpinene	1064	Sweet, Citrusy	n.d.	1.5 ± 0.5
Terpinolene	1088	Green	n.d.	2.0 ± 0
Linalool	1104	Fruity	4.0± 0	3.9 ± 0.4
Nonanal	1107	Piney	n.d.	1.0 ± 0.5
Ethyl 3-hydroxy-hexanoate	1138	Citrusy	2.6 ±1.0	n.d.
β-Terpineol	1146	Green	3.3 ± 0.6	n.d.
Citronellal	1156	Minty	n.d.	3.0 ± 0
NI	1167	Camphor, Minty	2.8 ± 0.5	n.d.
NI	1176	Green	n.d.	2.7 ± 1.0
Terpinen-4-ol	1186	Green	2.4 ± 1.0	n.d.
α-Terpineol	1196	Lemon, Piney, Minty	3.5 ± 0.5	3.3 ± 1.0
Decanal	1206	Fruity, Soap, Balsamic	3.7 ± 0.6	3.6 ± 0.5
NI	1213	Metal	2.6 ± 0.8	n.d.
*trans*-Carveol	1222	Caraway	2.6 ± 0.5	n.d.
Nerol	1232	Lemon	2.8 ± 0.4	n.d.
Neral	1244	Rose	1.3 ± 0.6	n.d.
Carvone	1250	Caraway	2.3 ± 0.5	n.d.
NI	1253	Fruity	n.d.	2.0 ± 0
Geraniol	1258	Floral, Lemon, Minty	2.6 ± 0.8	n.d.
NI	1259	Floral	n.d.	2.6±0.5
Geranial	1273	Floral, Citrusy	2.0±0	2.0±1.0
Perillaldehyde	1278	Green	2.3 ± 0.6	2.5±0.5
Thymol	1296	Metal	1.0 ± 0	n.d.
*trans,trans*-2,4- Decadienal	1323	Fatty	3.0 ± 0	2.2 ± 0.4*
α-Cubebene	1352	Herb	1.3 ± 0.5	n.d.
Geranyl acetate	1386	Sweet	1.2 ± 0.4	n.d.
β-Cubebene	1397	Fruity	n.d.	1.0 ± 0
Valencene	1499	Citrusy	2.8 ± 0.4	1.2 ± 0.4*
Bicyclogermacrene	1500	Green	n.d.	2.0±0
δ-Amorphene	1523	Fruity	2.0 ± 0.8	2.3 ± 1.0

NI: not identified by GC-MS; n.d.: not detected; * indicates significant difference (P<0.05) between the fruit juice and peel oil

A total of 31 components appeared to contribute to the aromatic profile in Jinchen fruit juice. Among them, ethyl butanoate, β-myrcene, octanal, linalool, and decanal decided the juice aroma because they are the strongest aroma active compounds perceived by all the panelists. Regarding the peel oil, 22 components appeared to contribute to the overall aroma. Linalool, α-pinene, and decanal defined the aroma of the oil, because these compounds had the greatest aroma intensity. Twelve compounds were perceived by all the panelists in both juice and oil. Among them, five aromatic compounds present different odor intensity in both samples, such as α-pinene, β-myrcene, octanal, (*trans, trans*)-2,4-decadienal, and valencene. The intensity of octanal, *trans, trans*-2, 4- decadienal, and valencene showed the significant difference (P<0.05) between the fruit juice and peel oil. Especially, valencene exhibited higher intensity in Jinchen fruit juice than in the peel oil, though valencene was not generally considered an important contributor to orange juice flavor in previously reports [20,21,22,23].

The different overall aroma between fruit juice and peel oil are attributed to the various composition of aroma active compounds [35]. The components which were perceived by all the panelists and only detected in the fruit juice or peel oil respectively may contribute to the aromatic differences. Among these, 19 components have been perceived only in the juice and 10 compounds were described as aromatic components of only the peel oil by the three panelists. Nineteen volatile components detected by the panelists only in fruit juice contribute to citrusy, green, and piney notes. The other ten volatile components described as aromatic components of only the peel oil impart a camphor, minty, green, fruity, and floral aroma.

According to these results, not all of the components found in greatest concentration by GC-MS contributed to the aroma in orange juice. Limonene was present in highest amount versus other volatiles, but did not exhibit strongest aroma activity though it has orange odor in GC-O of fruit juice and peel oil. In contrast, ethyl 3-hydroxyhexanoate and nerol were not detected by GC-MS and found in low concentration, contributing to citrusy and lemon aroma of the fruit juice according to GC-O intensity data.

Most odorant compounds characterized in Jinchen orange juice were also detected in some sweet orange juice [20,21], such as ethyl butyrate, myrcene, octanal, decanal, terpinen-4-ol, linalool, limonene, α-pinene, and hexanal, but nonanal and *(E)*-2-nonenal was not found to be major aroma active compound in Jinchen juice. There were also some aroma active compounds detected in Jinchen juice not commonly reported in other orange juices, such as ethyl 3-hydroxyhexanoate, perillaldehyde, thymol, *trans,trans*-2,4-decadienal, δ-amorphene. The aroma activities of these compounds in sweet orange flavor are not well documented. Most volatile compounds detected in Jinchen peel-oil were also found in many citrus oils, such as limonene, α-pinene, myrcene, octanal, decanal, terpinen-4-ol, linalool, γ-terpinene, terpiolene, citronellal, neral, α-copaene, valencene and nootkatone. These compounds were previously reported in other citrus oils but their aroma activities and contribution to citrus oil flavor were not described [9,10,11,36,37,38,39]. δ-Amorphene was the only compound found and identified tentatively in Jinchen peel-oil not previously reported in other citrus essential oils.

## Conclusions

In conclusion, in this study of the aromatic composition of fruit juice and peel oil of Jinchen orange a total of 57 components identified. GC-O was used to detect the aroma active compounds. There are 31 aromatic compounds present in the fruit juice, and 22 components appear to be the major contributors to the peel oil aroma, respectively. Twelve compounds were perceived by all panelists in juice and peel samples. Five compounds not identified by GC-MS were characterized in both samples. For fruit juice, ethyl butanoate, β-myrcene, octanal, linalool, and decanal had greatest aroma intensity. Linalool, α-pinene, and decanal presented highest aroma intensity in peel oil. Nineteen components have been perceived only in the juice sample, and ten compounds were described as aromatic components of only the peel oil by the panelists. These differences probably lead to the different overall aroma between fruit juice and peel oil. The aromatic composition in the fruit juice was more complex than that of the peel oil.

## Experimental

### Fruit juice and peel oil samples

Unpasteurized Jinchen orange fruit juice and peel oil were obtained from a local manufacturer of citrus products located at Songzi in Hubei province. Both the juice and the peel oil were derived from the same fruit source. The fruit juice was processed with a Polycitrus juice extractor. The peel oil was obtained from cold-pressed peel of the Jinchen orange fruit. The juice and oil samples were stored at ‑20 °C until analyzed.

### Extraction of Volatile Compounds

The manual SPME device equipped with a 50/30 μm DVB/CAR/PDMS fiber (Supelco, Bellfonte, PA, USA) was used for extraction volatile compounds of fruit juice. The fiber was conditioned in the GC injector port at 270 °C for 1 h prior to use. The orange juice (5 mL) and peel oil (5 mL) were placed into a 20 mL vial containing a micro stirring bar, respectively. The sample was equilibrated at 40 °C±1 °C for 15 min and headspace extracted by DVB/CAR/PDMS fiber for 40 min at the same temperature under stirring (500 rpm). After extraction, the fiber was inserted into the injection port of the GC to desorb the analytes for 5 min. Each analytical sample was measured in triplicate. The results are reported as the mean values of relative peak area percent ± SD (standard deviation)

### Chemicals

Authentic standards were obtained as follows: α-pinene were purchased from Aldrich Chemical Co. (Germany) *n*-alkanes (C_8_-C_20_), ethyl butyrate, β-myrcene, linalool, decanal, were obtained from FluKa Chemical Co. (Germany). Hexanal, *trans*-2-hexenal, benzaldehyde, octanal, limonene, nonanal, α-terpineol, neral, geranial, neryl acetate, geranyl acetate, citronellyl acetate and valencene were gifts from the GLD-Boton Essential Company (P. R. China).

### GC-MS analysis

GC-MS was carried out using a HP 5975B quadropole mass selective detector (Agilent Technologies, USA). The Mass spectral ionization temperature was set at 230 °C. The mass spectrometer was operated in the electron impact ionization mode at a voltage of 70 eV. Mass spectra were taken over the *m/z* range 30–400.

The flow rate of the helium carrier gas on HP-5 column (30 m×0.25 mmI.D, 0.25 μm film thickness, J&W Scientific, Folsom, CA, USA) was 1 mL/min. The analysis performed in the splitless mode and injector temperature was 250 °C. The column was held at 40 °C for 3 min, and then increased from 40 °C to 160 °C at 3 °C/min, held at 160 °C for 2 min, and finally increased to 220 °C at a rate of 8 °C/min, then held for 3 min.

Volatile components were identified by comparing their mass spectra with the mass spectra from MS libraries (NIST 05, WILEY 7.0). When available, MS identifications were confirmed by comparing GC retention times of the analytes with those from pure standards. The identification was confirmed using retention indices (RI), and the value was compared with those reported in the literature. Linear retention indices (RI) of the compounds were calculated using a series of *n*-alkanes (C_8_–C_20_, Sigma-Aldrich, Germany) injected in the same conditions. When standard chemicals were not available, tentative identification was carried out by matching the mass spectra. The results are given in Table 1.

### GC-Olfactometry

The analysis was carried out using a HP 6890 GC equipped with a HP-5 column (30 m×0.25 mm I.D, 0.25 μm film thickness, J&W Scientific, Folsom, CA, USA) directly connected to a HP 5975 series mass selective detector and a sniffing port (ODP2, Gerstel,Inc., Baltimore, MD). The GC effluent was split 1/2 between the mass spectrometer and the sniffing port. The transfer line to the GC-O sniffing port was held at 280 °C. The volume of sample extract analyzed and oven program temperatures were the same as those described above for the GC-MS. Humidified air was added in the sniffing port at 60 mL/min. Compound identifications were made by comparison of the mass spectra, retention times, and the RI of the volatile components in both extracts with those of corresponding reference standards. The mass spectrometer retention times were compared with the retention times measured in the olfactometry runs, and both were compared to reference standards.

Three panelists were used for the intensity detection and verbal description of the odor active components identified in both extracts by GC-O. The first training was familiarization with the scale and the system in a short period. During this period the panelists performed three times analysis of one of the test samples and rate intensity of the eluted odor using the four scale (1=extremely weak, 2=clear but not intense odor, 3=intense odor, 4=extremely strong). They were not instructed about what was the expected intensity of the odors and each one used the intensity category, which in his / her opinion fitted best the intensity of the odor perception caused by the eluted odorant. Test data were recorded only after panelists demonstrated an ability to replicate analyses and the scales given by each panelist were not significant difference. After the successful test, the trained panelists carried out sniffing the samples and recorded the perceived intensities of the odor.

The results of intensity from two samples were statistically evaluated by one way analysis of variance (ANOVA). Statistically, differences with P-values under 0.05 were considered significant
